# The enemy within: the association between self-image and eating disorder symptoms in healthy, non help-seeking and clinical young women

**DOI:** 10.1186/s40337-015-0067-x

**Published:** 2015-08-25

**Authors:** Emma Forsén Mantilla, Andreas Birgegård

**Affiliations:** Karolinska Institute, Department of Clinical neuroscience, Resource center for eating disorders, Norra Stationsgatan 69, plan 7, 113 64 Stockholm, Sweden

**Keywords:** Eating disorders, Self-image, Interpersonal theory, Age, Non help-seeking

## Abstract

**Background:**

Previous research has shown self-image according to the interpersonal Structural Analysis of Social Behavior model, to relate to and predict eating disorder symptoms and outcomes.

**Methods:**

We examined associations between self-reported self-image and ED symptoms in three groups of 16–25 year old females: healthy (*N* = 388), non help-seeking (*N* = 227) and clinical (*N* = 6384). Analyses were divided into age groups of 16–18 and 19–25 years, and the patient sample was divided into diagnostic groups.

**Results:**

Stepwise regressions with self-image aspects as independent variables and eating disorder symptoms as dependent showed that low self-love/acceptance and high self-blame were associated with more eating disorder symptoms in all groups, except older patients with bulimia nervosa where self-hate also contributed. Associations were generally weaker in the healthy groups and the older samples.

**Conclusions:**

We put forward that older age, low desirability of symptoms, poorly working symptoms, and being acknowledged as ill, may weaken the association, with implications for treatment and prevention.

## Background

### Eating disorders and interpersonal functioning

There is a growing body of research focusing on interpersonal difficulties in people with eating disorders (ED). Specific attention has been devoted to attachment, social and affective communication and the perception of self and others [1, 2], with ED populations consistently displaying patterns of insecure attachment, impaired interpersonal skills and negative self-image compared to normal controls and other clinical groups. These factors are likely to be connected to the disorder, but it is unclear if they are a result or a cause. Most previous research has however identified these deficits as risk- or maintaining factors [3–7].

### Theoretical model: the structural analysis of social behavior

**Fig. 1:**
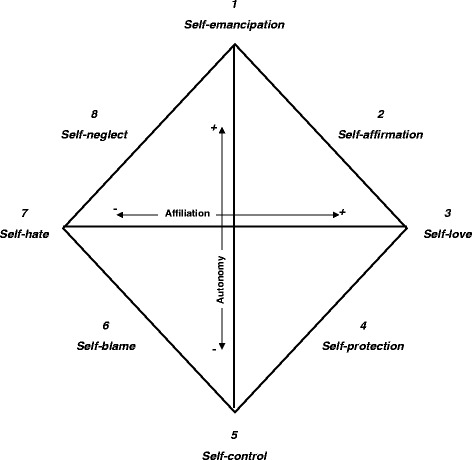
The SASB Introject cluster model. The model displays the eight clusters and the two axes (Affiliation and Autonomy). From: Benjamin, L.S. (1996). Interpersonal diagnosis and treatment of personality disorders, 2nd Ed. N.Y.: The Guilford Press [13]. © The Guilford Press

The Structural Analysis of Social Behavior, SASB [8, 9], is a model based on interpersonal- and attachment theory, and encompasses attachment behaviors, interpersonal behaviors and self-image. According to interpersonal theory [10], a persons’ self-image is formed in interaction with primary attachment figures and it influences subsequent interpersonal behavior. In the model, interpersonal behaviors are organized around two dimensions: Affiliation (love vs. hate) and Autonomy (control vs. autonomy). In the model there are three surfaces, each representing a specific focus of interpersonal behavior: surface 1, focusing on another person (transitive focus), surface 2, focusing on own reactions (intransitive focus) and surface 3, internalized actions towards oneself (introjection). Figure 1 shows the introject or self-image surface of the model, measuring self-treatment or self-directed behavior, with the two dimensions represented horizontally and vertically. Points along the perimeter represent combinations of the two underlying dimensions and form eight clusters. SASB self-image variables have been shown empirically to relate to attachment and social behavior in previous research, e.g. [11, 12].

### Self-image and ED

ED patients’ self-image profiles are diagnostically distinct and significantly more negative than controls [14]. A negative self-image has been identified as a risk factor for ED, e.g. [15, 4], is associated with poor outcome [16] and predicts dropout from treatment [17]. In a previous study [18], we found an association between ED symptoms and negative self-image in a sample of healthy, young adolescent girls. More specifically, self-blame and, negatively, self-affirmation related to symptoms typical of ED (body dissatisfaction, eating concern, food restriction etc.). The same association, but more than twice as strong, was observed in our clinical sample of girls. ED symptoms thus seem central for self-acceptance (and/or vice versa) in both healthy girls and girls with ED.

In our previous study [18], we speculated that ED symptoms could shape and be shaped by introject variables, due to the ED mimicking being treated critically by a significant other. People tend to seek confirmation of their self-image, whether positive or negative, in interactions with others [11]. Having been treated critically might then increase susceptibility to ED, since ED symptoms in themselves would be an extension of such interactions. Subsequently, ED-related increased rejection and non-acceptance of oneself increase self-criticism generally, analogous, again, to being treated thus by an important other. In order to further investigate associations between self-image and ED symptoms, we wished to extend upon previous results through examining normal and clinical older adolescents and young adults, and also including a non help-seeking sample with high ED symptom load.

### The present study

The association between ED symptoms and self-image aspects is potentially important when attempting to understand the complex etiology of ED and its psychological mechanisms. It might also contribute knowledge regarding who is at risk of developing an ED, and can give important hints about effective ways of interacting with ED patients in treatment, since SASB self-image has direct and documented associations with interpersonal behavior. The samples in our previous study were relatively young (13–15). Studying older samples may inform about the development of the association with increasing age. Mean age of ED onset ranges between 15–17.1 depending on diagnosis [19], and incidence rates are highest for females aged 15–24 [20]. Also, many transitions happen in mid-adolescence (physical, sexual, psychosocial etc.), which may influence both self-image and ED symptoms. Furthermore, ED in young adolescents is predominantly restrictive (anorexia nervosa, AN, or restrictive type atypical presentations), with relatively few cases of bulimia nervosa (BN) or other binge-purge patterns. Studying older groups thus allows for broader diagnostic comparisons, which is important as previous research has demonstrated differences in the psychological profiles of the different disorders [21–24], including differences between AN and BN in terms of which aspects of self-image predict outcome [25]. Young age and suffering from AN also tends to be associated with denial of illness and a lower desire for help [26]. Such factors potentially influence the association between ED and self-image aspects, and therefore further warrants contrasting age- and diagnostic groups.

In addition to healthy individuals and individuals with ED, there is a large group of individuals (mainly girls) who experience sub-clinical ED problems. The prevalence of sub-clinical ED in young adult females is around 8.5 % [27], while prevalence rates for full-syndrome EDs is between 0.4 - 7.7 % depending on diagnosis [27–30]. Some sub-clinical individuals go on to develop full-blown EDs and others do not, but experiencing sub-threshold ED symptoms (dieting, body dissatisfaction, negative self-esteem) puts them at higher risk of developing an ED [31, 32]. Further, there is an estimated large population of unrecorded cases who do not seek help, i.e. who suffer from full ED but are not in treatment [33]. It is important to learn more about subclinical and non help-seeking groups, since this may inform prevention and outreach efforts (what to target, when to target it) and could provide clues about who is at risk of developing an ED; we term this group non help-seeking henceforth since our data suggested similar symptom levels to the clinical groups (see below). Also, looking at psychological profiles in terms of self-image in highly symptomatic people who are and are not in treatment, may give clues as to who is likely to seek help and who is not.

### Aims

In this study we aimed to examine and compare associations between different aspects of self-image and ED symptoms in healthy-, non help-seeking, and clinical 16–25 year old females. As the age range is large and as there may be variations due to age, we examined the younger groups (16–18) and the older groups (19–25), separately. Based on previous findings, we hypothesize that the associations between ED symptoms and self-image aspects will be stronger in the clinical groups than in the healthy groups. As far as the non help-seeking groups go, we make no a priori assumptions, as the association between ED and self-image has not been investigated in such a group before. Within the clinical sample, we also look at three distinct diagnostic categories (AN, BN and ED not otherwise specified, EDNOS) within each age group. Contrasting the samples may help distinguish psychological aspects typical of the different groups.

## Methods

### Participants

#### Healthy sample

For the age group 16–18 year olds, data was collected at three selected high schools in the Stockholm region. All students aged 16 and older were invited to participate. Out of a possible 705 females, 207 (30 % response rate) completed the questionnaires. Three individuals (0.4 %) were excluded due to being outside the age group and one (0.1 %) due to incomplete data. This left 203 participants (29 %) with a mean age of 16.7 (*SD* = .62). Participants, aged 19–25, were recruited at Stockholm University; of 251 potential participants, 193 (76.9 %) students completed the questionnaires. Eight (3.2 %) were excluded due to missing data, resulting in *N* = 185 (74 %) with a mean age of 22.0 (*SD* = 1.84).

### Non help-seeking sample

Participants were recruited via advertisements online and in a newspaper, and 138 (61 % of the sample) in the age range 16–18 (*M* = 16.8, *SD* = .75) and 89 (39 %) in the age range 19–25 (*M* = 21.2, *SD* = 1.77) completed the questionnaires. This recruitment was originally intended to produce a subclinical sample, but symptom levels (see Results) indicate that it is in fact better construed as an ill sample not in treatment.

### Clinical sample

Data came from Stepwise, a large-scale naturalistic quality assurance database and data collection system for specialized ED treatment units (*N* = 26) in Sweden [34]. Inclusion criteria are medical- or self-referral to one of the treatment units, a DSM-IV ED diagnosis, and intention to treat the patient. At the time for data extraction (19^th^ of March, 2015), there were 7542 patients in the age range 16–25. Out of these, 290 (3.8 %) participants were excluded due to lack of consent to research participation, 7 (.1 %) were excluded due to incomplete registration, 199 (2.6 %) were excluded due to lack of ED diagnosis, 358 (4.7 %) diagnosed with "EDNOS Other" were excluded due to the unspecific nature of the diagnosis (also, this group is unlikely to belong to the ED population; [35]), as were 231 (3.3 %) males and 73 (1.0 %) participants with missing data. The remaining sample comprised 6384 (85 %) female patients, of whom 2295 (36 %) were 16–18 years old and 4089 (64 %) were 19–25.

### Instruments

#### Eating Disorder Examination Questionnaire (EDEQ)

The EDEQ is a 36 item self-report measure [36] used to measure eating pathology. Items focus on the past 28 days and are rated on a 0–6 scale, except for frequencies of key ED behaviors, which are assessed in terms of number of occurrences over the past 28 days. The instrument provides a global score and four subscale scores: Eating concern, Shape concern, Weight concern and Restraint. It is a commonly used instrument and has good psychometric properties and reference data [36–39]. In this study all subscales had acceptable internal consistency in all samples and age groups with Cronbach’s alphas ranging between .69 and .92. Cronbach’s alpha for the global scale, used in analyses below, was > .70 throughout.

### Structural Analysis of Social Behavior (SASB)

This is a 36 item self-report measure assessing self-image in terms of the SASB model. It divides into eight cluster variables; 1) Self-emancipation, 2) Self-affirmation, 3) Self-love, 4) Self-protection, 5) Self-control, 6) Self-blame, 7) Self-hate, and 8) Self-neglect. Items are rated on a 0 to 100 scale indicating increasing levels of agreement. The original instrument has good internal consistency with Cronbach’s alpha = .76 [9], as does the Swedish translation with alpha = .87 [40]. SASB discriminates well between psychiatric diagnoses [9, 41] including between ED, [14] and factor analyses confirm the underlying model [8, 9]. In the present study clusters were excluded when Cronbach’s alphas where < .70 for both age groups in either sample. Six out of eight clusters yielded acceptable alphas (Self-affirmation, Self-love, Self-protection, Self-blame, Self-hate and Self-neglect) in both age groups in the high-risk and clinical sample, but as Self-neglect did not reach acceptable alpha in either age group of the normal sample, five clusters remained to be analyzed. In order to check our findings, analyses were however also repeated including all clusters (data not shown), but no substantial changes to the results occurred.

### Structured Eating Disorder Interview (SEDI)

The SEDI was the semi-structured interview used to determine DSM-IV ED diagnoses and subtypes in the clinical sample. Patients are assessed with between 20 and 30 questions depending on which criteria that are considered fulfilled. It has good concordance with the EDE interview concerning ED diagnosis (81 %, and Kendall’s Tau-b of .69, *p* < .001; [42].

### Procedure

#### Healthy sample

Regarding the 16–18 year olds, letters were sent to parents informing about the aim and procedure of the study. Parents were encouraged to contact the project supervisor if they had questions. Students were primarily informed about the study by their teachers, but there was also information available on the schools’ intranet and on posters around the schools. They were informed that the study concerned self-image and eating- and shape concerns. The questionnaires were filled out via a secure online connection during school hours and all participants gave their informed consent. They were also told that participation was voluntary and confidential. Completion of the questionnaires took approximately 30 minutes. The teachers and the student health care team were well informed about the study in case filling out the forms would cause worry or concern.

The 19–25 year olds were recruited in lectures, via ads around the university or via drop-in on-site (at a university department). The occasions for drop-in were announced by bulk email and on notice boards. All participants were told that participation was voluntary and confidential. Before completing the questionnaires they signed informed consent. Time for participation was about 30–40 minutes. Participants recruited in lectures received the questionnaires and a postage paid envelope, those who responded to ads emailed their address and were sent the materials, and those who dropped in completed the forms on site. All participants were rewarded either by gift certificate (approx. 15 USD) or course credit.

### Non help-seeking sample

Advertisements were posted online (Facebook and relevant webpages) and in a Stockholm based newspaper. The ads called for participants in the age range 16–25 with some concerns about their shape and weight and with a wish to improve their self-esteem. Individuals who wished to participate received an email with login details and they completed the questionnaires via a secure online connection. Prior to completing the forms they gave their informed consent and had to state whether they were in, or had had, any type of treatment for eating related issues. If they stated that they had been, or were in ED treatment, they were not able to participate. This was to ensure the sample comprised high-risk individuals and not individuals with full-blown EDs. Participants were offered a place in an ED prevention program if judged to be at-risk for developing an ED, or advised to seek ED treatment if judged too ill for the prevention intervention. In terms of symptom severity, this group may have consisted of both subclinical and ill but non help-seeking individuals, but overall, symptom levels appeared to be well within a clinical range (see Table 1).

**Table 1:** Means and standard deviations for SASB clusters and EDEQ in all groups

Subscales	Normal sample (N = 388)		Non help-seeking sample (N = 227)		Clinical sample (N = 6384)							
	16-18 (N = 203)	19-25 (N = 185)	16-18 (N = 138)	19-25 (N = 89)	16-18 (N = 2295)				19-25 (N = 4089)			
					Total	AN (N = 702)	BN (N = 427)	EDNOS (N = 1166)	Total	AN (N = 865)	BN (N = 1328)	EDNOS (N = 1896)
	m (sd)	m (sd)	m (sd)	m (sd)	m (sd)	m (sd)	m (sd)	m (sd)	m (sd)	m (sd)	m (sd)	m (sd)
SASB 2	67.7 (22.9)	63.4 (21.0)	39.8 (23.5)	32.1 (21.7)	34.1 (22.2)	36.5 (23.8)	29.8 (20.1)	34.3 (21.6)	28.5 (19.3)	30.6 (20.6)	26.8 (18.6)	28.8 (19.1)
SASB 3	63.9 (20.6)	63.8 (18.9)	36.1 (22.6)	33.2 (20.9)	33.4 (21.1)	34.5 (22.1)	30.6 (19.9)	33.8 (20.9)	30.5 (19.6)	30.7 (20.4)	29.1 (18.9)	31.4 (19.6)
SASB 4	64.2 (18.9)	65.4 (15.8)	42.6 (19.8)	39.8 (18.9)	45.0 (20.0)	47.6 (20.3)	40.9 (19.0)	44.9 (19.9)	40.9 (19.5)	41.3 (20.0)	39.8 (19.0)	41.4 (19.5)
SASB 6	22.0 (19.2)	30.1 (21.9)	51.2 (26.0)	56.5 (24.1)	52.9 (24.6)	51.0 (25.4)	59.2 (23.4)	51.7 (24.2)	57.7 (23.0)	57.8 (24.0)	60.2 (21.9)	56.0 (23.1)
SASB 7	15.2 (16.7)	18.0 (17.6)	38.7 (27.4)	40.8 (26.6)	40.7 (25.2)	39.8 (26.2)	47.8 (24.6)	38.6 (24.4)	44.7 (24.2)	46.5 (25.1)	47.3 (23.7)	42.0 (23.9)
EDEQ Global	1.70 (1.4)	1.73 (12.0)	3.56 (1.3)	3.94 (1.2)	3.6 (1.4)	3.27 (1.5)	4.25 (1.1)	3.56 (1.4)	3.90 (1.2)	3.59 (1.4)	4.18 (1.0)	3.84 (1.2)

*SASB* Structural Analysis of Social Behavior, *SASB 2* Self-affirmation, *SASB 3* Self-love, *SASB 4* Self-protection, *SASB 6* Self-blame, and *SASB 7* Self-hate, *EDEQ* Eating Disorder Examination Questionnaire

### Clinical sample

ED professionals assessed the patients using Stepwise. Stepwise assessment is performed within the patient’s third visit to the treatment unit and takes around 45 minutes. Prior to the assessment, patients receive information about stepwise and about research participation being voluntary. The assessment starts with the Structured Clinical Interview for DSM-IV Axis 1 disorders (SCID; [43]) followed by the SEDI, clinical ratings of level of functioning and ED severity and ends with self-report measures (EDEQ and SASB, followed by other instruments measuring psychiatric symptoms not considered here). During the first part of the assessment the clinician is seated at the computer recording the answers on screen with the patient sitting opposite. When filling out the self-report measures the patient sits at the computer and the clinician usually leaves the room. The Stockholm Regional Ethics Review board has approved this study (2013/82-31/4).

### Statistical analysis

We present descriptive statistics on SASB clusters and EDEQ subscales and global scale in order to show how these variables varied in the different groups. The skewness of all variables was checked prior to analyses, and for variables with skewed distributions, logarithms were calculated. In the normal sample (both age groups), the logarithm of SASB cluster 7 had to be used and in the clinical 16–18 year olds with a BN diagnosis, the logarithm of SASB cluster 2 had to be used. All other variables displayed adequately normal distributions in all groups. The results were analyzed using stepwise regression with EDEQ global score as dependent variable and the SASB clusters as independent variables. We used a forward selection procedure in which, based on the p-value of F, the independent variable with the smallest p-value is entered into the model one at a time. This process is repeated until no further improvement of the model is possible. Prior to the analyses, bivariate outliers were defined as observations with jack-knife residuals beyond the critical *t* for *p* < .01, which controls for different group sizes and number of predictors. Jack-knife residuals are studentized deleted residuals distributed as *t* with *df* = *n* – *k* - 2, where *k* is the number of predictors [44]. This resulted in elimination of between 0.7 and 4.2 % of the participants in the different groups. Outliers were removed consecutively from the models before the Stepwise regressions were computed. With large sample sizes in our clinical groups, we risked over inclusion of predictor variables in the regression models, and we therefore only report variables contributing more than 1 % independent variance to the models and that are significant at the 0.001 level (0.01 for the healthy and non help-seeking samples due to smaller sample sizes).

## Results

### Between-group comparisons on EDEQ and SASB

To illustrate variations between groups regarding self-image and ED symptoms, descriptive data is presented in Table 1. The lowest EDEQ Global scores were, unsurprisingly, observed in the healthy groups. These groups also displayed largely positive self-images. The non help-seeking groups and the clinical groups were similar in their EDEQ scores and their self-image scores. Comparing diagnostic groups, the BN groups had the most negative self-image and the highest EDEQ scores.

### Associations between SASB clusters and ED symptoms: healthy sample

Stepwise regression analysis showed that for the 16–18 year olds, Self-affirmation alone was associated with EDEQ score and explained 35 % of the variance (Table. 2). In the 19–25 age group Self-blame exclusively was associated with EDEQ scores; no other clusters contributed to the model (Table 2). The explained variance in the relationship between self-image and ED symptoms was larger in the 16–18 age group (35 %), than in the 19–25 age group (30 %) but the difference between these was not significant.

**Table 2:** Stepwise regression results using SASB cluster subscales to predict ED symptoms: healthy and non help-seeking sample

Models	r^2^	R^2^	t	p	β
Healthy sample					
16–18 year olds					
Step 1: Self-affirmation	.36	.35	−9.27	<.001	-.60
19-25 year olds					
Step 1: Self-blame	.30	.30	8.06	<.001	.55
Non help-seeking sample					
16-18 year olds					
Step 1: Self-affirmation	.45	.45	−4.93	<.001	-.40
Step 2: Self-blame	.53	.52	4.72	<.001	.39
19-25 year olds					
Step 1: Self-love	.46	.45	−3.59	.001	-.39
Step 2: Self-blame	.53	.52	3.57	.001	.39

### Associations between SASB clusters and ED symptoms: non help-seeking sample

In the 16–18 age group, low Self-affirmation and high Self-blame were associated with ED symptoms (Table 2), contributing almost equally to the model. Among 19–25 year-olds, Self-love and Self-blame contributed significantly and equally to the model (Table 2). The association between ED symptoms and self-image was substantial in both age groups within this sample with 52 % of the variance explained in both models and no significant difference between the groups.

### Associations between SASB clusters and ED symptoms: clinical sample

Within this sample stepwise regressions were carried out for each age group and each diagnostic category separately. In the 16–18 group as a whole, three variables explained a total of 55 % of the variance in EDEQ scores (Table 3): Self-blame positively, and Self-affirmation and Self-love negatively. In the young AN group, Self-blame and Self-affirmation explained 61 % of the variance in the expected directions*.* In the young BN group, the explained variance was 39 % and contributing most Self-love and Self-blame contributed most. Finally, in the young EDNOS group, 53 % of the variance was explained by Self-blame and Self-love in the expected directions.

**Table 3:** Stepwise regression results using SASB cluster subscales to predict ED symptoms: clinical sample, 16–18

Models	r^2^	R^2^	t	p	β
Full age group					
Step 1: Self-blame	.46	.46	20.77	<.001	.40
Step 2: Self-affirmation	.54	.54	−9.16	<.001	-.23
Step 3: Self-love	.55	.55	−7.57	<.001	-.19
AN					
Step 1: Self-blame	.54	.54	14.03	<.001	.48
Step 2: Self-affirmation	.61	.61	−10.85	<.001	-.37
BN					
Step 1: Self-love	.32	.32	−8.20	<.001	-.39
Step 2: Self-blame	.40	.39	6.71	<.001	.32
EDNOS					
Step 1: Self-blame	.45	.45	16.12	<.001	.43
Step 2: Self-love	.53	.53	−13.88	<.001	-.37

Examining the 19–25 group overall, three variables explained 42 % of the variance in EDEQ scores (Table 4): Self-blame positively, and Self-love and Self-affirmation negatively. In the AN group in this age range, Self-love and Self-blame explained 52 % of the variance in the expected directions. For the BN group, three clusters contributed significantly to the model: Self-blame and Self-hate positively and Self-love negatively. Together they explained 37 % of the variance. In the EDNOS group, three variables explained 39 % of the variance: Self-blame, Self-love and Self-affirmation in the expected directions. We further directly compared the full model *R*s of the different diagnostic categories and age groups using a *z*-test [45], and all differences were significant with *p*s < .05, except between the two BN groups and between the BN and EDNOS groups within the 19–25 group.

**Table 4:** Stepwise regression results using SASB cluster subscales to predict ED symptoms: clinical sample, 19–25

Models	r^2^	R^2^	t	p	β
Full age group					
Step 1: Self-blame	.34	.34	21.10	<.001	.33
Step 2: Self-love	.41	.41	−10.81	<.001	-.22
Step 3: Self-affirmation	.42	.42	−8.95	<.001	-.18
AN					
Step 1: Self-love	.46	.46	−13.07	<.001	-.44
Step 2: Self-blame	.52	.52	10.11	<.001	.34
BN					
Step 1: Self-blame	.30	.30	8.75	<.001	.29
Step 2: Self-love	.36	.36	−9.30	<.001	-.26
Step 3: Self-hate	.37	.37	4.79	<.001	.16
EDNOS					
Step 1: Self-blame	.31	.31	13.32	<.001	.31
Step 2: Self-love	.38	.38	−7.16	<.001	-.21
Step 3: Self-affirmation	.39	.39	−6.46	<.001	-.19

## Discussion

The study tested whether different aspects of self-image as measured by the SASB, were associated with ED symptoms in healthy, non help-seeking and clinical 16–25 year old females. Blame-criticism and/or love-acceptance best explained variance in ED symptoms in the expected direction in all samples and groups, with the exception of the 19–25 year old BN patients where hate also made an important contribution. Associations were weaker in the healthy groups and the older samples (not true for the non help-seeking 19–25 year olds though), but all associations were nevertheless considerable.

### Healthy sample

The association between self-image and ED symptoms for our healthy females looked similar to our previous results with healthy 12–15 year old girls [18]. The association was strongest in the 16–18 age group (compared to both the younger girls in our previous study and the 19–25 year olds in this study), suggesting a slightly elevated risk of valuing oneself based on bodily appearance at this age. Indeed, levels of thin-ideal internalization seem to increase throughout adolescence [46]. Also, the most common age of ED onset is within the 16–18 age range [19]. A previous study has shown that in a sample of 10–18 year old females, the preference for a thinner than average body shape, body dissatisfaction and ED symptoms increased with age [47]. Furthermore, in a population-based longitudinal study, a decrease was found in the prevalence of ED related behaviors (compensatory behaviors, binge eating) from age 14–16 years to 23 years [48]. Also, self-esteem tends to become more stable, less contingent and higher with increasing age [49] and the most significant change in self-esteem happens during the first decade of young adulthood [50]. Hence, a weaker association between self-acceptance/self-criticism and ED symptoms seems reasonable in a normal, older age group.

### Non help-seeking sample

In this sample, self-image aspects were associated with ED symptoms strongly regardless of age. These individuals appear to value themselves almost exclusively in terms of how well they succeed in their quest to restrict food intake and control weight and shape. At this stage of ED, which for some may be subclinical but for most mean quite serious illness levels without seeking treatment, it might be that they have not yet experienced or ignore negative consequences of their behaviors and instead embrace such behaviors as, at least partly, helpful and positive; sometimes referred to as the "honeymoon phase" of ED. Research has demonstrated that denial and concealment of ED behaviors is present in the early stages of illness [51] and although aware of EDs, these individuals strongly believe it does not apply to them [52]. Additionally, research has indicated that unless women with ED have identified problems with their behaviors (psychological distress, interference with life, health problems etc.), they are unlikely to seek help [53], and even when recognizing the ED as a problem, perceived benefits may decrease motivation to seek treatment [54]. As the illness progresses however, self-awareness and the recognition of the ED as something negative and destructive tend to increase, and eventually make individuals more ready to seek treatment [55, 56]. In the study by Vandereycken and Van Humbeeck [52] the participants furthermore reported that if a clinician had acknowledged that they had an ED earlier, they too would have accepted that, suggesting that a professional judgment may be critical in order to recognize ED symptoms as problematic.

Our non help-seeking individuals seem to be at a stage where their self-image is strongly contingent on ED attitudes and behaviors (or vice versa) and therefore still denying the problematic nature of such behaviors. To illustrate; the more self-critical a person is, the more she needs her symptoms (in order to, e.g., regulate emotions and attempt reaching ideals of perfection and self-discipline) and the more she needs her symptoms and behaves accordingly, the more self-critical she becomes. This vicious circle inherently prevents the person from acknowledging this as problematic. Another possibility is that these individuals lack a social network motivating and helping them get in touch with a treatment unit. Previous research has shown that interpersonal feedback showing concern for the individual and encouragement from friends and family, are important positive contributors in the help-seeking process [15, 53, 57, 55].

### Clinical sample

The relationship between acceptance/criticism and ED symptoms was strongest in the 16–18 year olds, yet it was weaker than for the clinical group in our previous study (*R*^*2*^ = .64). The weakest association was observed in the 19–25 age group and this relationship was even weaker than that of the non help-seeking 19–25 year olds. Perhaps here is a benefit, not only from older age, but also from being acknowledged by ED professionals as ill; getting older and realizing one's behaviors are problematic and considered an illness, potentially reduce the meaning of the symptoms to the persons self-image. Consistent with this, both BN groups scored highest on EDEQ and therefore had weaker associations compared to the other diagnostic groups; more subjective suffering from ED was associated with a more detached self-image. The young AN group showed the opposite pattern, with the lowest EDEQ score but the strongest association overall, suggesting the link between self-image and bodily appearance is exceptionally strong. As reasoned above, self-image may be more associated with valued ED symptoms because they, at least partially, work for the individual. Cognitive dissonance theory [58] assumes we seek harmony between our attitudes, beliefs and behaviors, and if that is not achieved, one or the other needs to be altered. Patients with BN may e.g. be unable to integrate binge-eating (behavior) into their self-image as it goes against their pursuit of the thin-ideal (belief), whereas AN patients have less difficulty integrating their predominantly restrictive eating into their self-image since the behavior works toward the overvalued thin-ideal. As patients become older and sometimes migrate to other, less restrictive diagnoses [59], this consistency is jeopardized and cognitive dissonance allows previous positive beliefs about ED to change. Research has indeed shown that compared to AN patients, BN patients are more ready to change once they enter treatment [60, 61], and can improve significantly already during the first four weeks of treatment [62].

### General discussion

The association between ED and self-image differed not in form, but greatly in magnitude between groups, and this is true also if the pattern is extended to our previous study on younger participants; [18]. We have put forward that older age, being acknowledged as ill, low desirability of symptoms and poorly working symptoms, seem to weaken the relationship between self-image and ED symptoms. But why this association arises in the first place, we can only speculate. Sociocultural values such as the thin-ideal, and parental values, behaviors and attachment style lay the grounds for self-evaluation. Insecure attachment, low parental care, overprotective parental bonding, negative peer influence and internalization of the thin-ideal, are some of the established precursors of low self-esteem, self-criticism, perfectionism, body dissatisfaction and dieting [6, 46, 63–68], which are all in turn documented risk factors for later development of ED [31, 69–71]. As suggested above, according to interpersonal theory, an individual may be vulnerable to ED partly because that way of treating oneself is consistent with aspects of the person’s self-image [10]. The self-image in turn, mirrors the way the person has been treated in early significant relationships [8, 11], hence the ED mimics a significant other in the sense that it continues to reinstate the already negative self-image and therefore maintains its hold. Depending perhaps on how important this fictional “significant other” is deemed to be, its influence on the person’s self-image varies; when the ED is the most vital relationship one has, it will have an undue impact on the self-image (and vice versa).

### Limitations

The main constraint with this study is the cross sectional design; all conclusions inferring causality and/or progression over time require longitudinal data and must be considered with caution. Another drawback is the use of self-report measures: a certain degree of introspective abilities is needed in order to complete the questionnaires and this may vary between groups. Also, the conditions for completing the questionnaires differed between groups and this could potentially affect their answers. Further, response rate was low especially for the healthy younger sample, and could not be calculated for the non help-seeking sample. The latter sample, also, was partly defined post hoc based on the data rather than procedure (i.e., denial of receiving or having received ED treatment, but clinical range EDE-Q scores). A final drawback is that three of the eight SASB clusters (self-emancipation, self-control and self-neglect) did not yield acceptable Cronbach’s alphas and hence were excluded from analysis. This could be a problem specific to our samples or potentially a problem related to the Swedish translation of the instrument. However, the instrument has been successfully used in previous studies, yielding meaningful results in relation to the above-mentioned clusters (e.g. [25]). Also, as noted earlier, repeating analyses including all clusters did not change our results.

For these reasons, generalization must be cautious, and targeted replication with higher response rate is important.

## Conclusions and implications

Consistent with Forsén Mantilla and colleagues [18], we establish strong associations between certain aspects of self-image and ED symptoms in healthy and clinical girls. We also expand on previous work as we look at older cohorts and include a sample of non help-seeking but probably quite ill individuals. Our findings have implications for treatment of ED. For example, for young AN patients, where the association between self-image and symptoms is very strong, this needs to be thoroughly addressed and explored in the beginning of treatment. Both advantages and disadvantages of the illness need exploration, and the difficulty of changing something so fundamental for one's self-image as the ED, needs to be recognized. In order to decrease self-criticism and increase self-acceptance, these individuals should be met with openness, patience, empathy and acceptance rather than negative control, criticism and blame. As treatment progresses, the potential sense of loss of one's ED and the accompanying grieving process, also deserves attention and respect.

As acceptance/love and/or criticism are associated with ED symptoms in all groups, these aspects need attention not only in ED treatment, but also in prevention efforts. That the non help-seeking groups have the same strong associations, independent of age, is interesting and needs further examination as it may provide additional knowledge about early intervention or outreach efforts.
